# Asynapsis and unreduced gamete formation in a *Trifolium* interspecific hybrid

**DOI:** 10.1186/s12870-021-03403-w

**Published:** 2022-01-03

**Authors:** Helal A. Ansari, Nicholas W. Ellison, Isabelle M. Verry, Warren M. Williams

**Affiliations:** AgResearch Grasslands Research Centre, Tennent Drive, Palmerston North, 4442 New Zealand

**Keywords:** Unreduced gametes, Asynapsis, Meiotic nonreduction, *Trifolium*, Interspecific hybridization, Polyploidization

## Abstract

**Background:**

Unreduced gametes, a driving force in the widespread polyploidization and speciation of flowering plants, occur relatively frequently in interspecific or intergeneric hybrids. Studies of the mechanisms leading to 2*n* gamete formation, mainly in the wheat tribe *Triticeae* have shown that unreductional meiosis is often associated with chromosome asynapsis during the first meiotic division. The present study explored the mechanisms of meiotic nonreduction leading to functional unreduced gametes in an interspecific *Trifolium* (clover) hybrid with three sub-genomes from *T. ambiguum* and one sub-genome from *T. occidentale*.

**Results:**

Unreductional meiosis leading to 2*n* gametes occurred when there was a high frequency of asynapsis during the first meiotic division. In this hybrid, approximately 39% of chromosomes were unpaired at metaphase I. Within the same cell at anaphase I, sister chromatids of univalents underwent precocious separation and formed laggard chromatids whereas paired chromosomes segregated without separation of sister chromatids as in normal meiosis. This asynchrony was frequently accompanied by incomplete or no movement of chromosomes toward the poles and restitution leading to unreduced chromosome constitutions. Reductional meiosis was restored in progeny where asynapsis frequencies were low. Two progeny plants with approximately 5 and 7% of unpaired chromosomes at metaphase I showed full restoration of reductional meiosis.

**Conclusions:**

The study revealed that formation of 2*n* gametes occurred when asynapsis (univalent) frequency at meiosis I was high, and that normal gamete production was restored in the next generation when asynapsis frequencies were low. Asynapsis-dependent 2*n* gamete formation, previously supported by evidence largely from wheat and its relatives and grasshopper, is also applicable to hybrids from the dicotyledonous plant genus *Trifolium*. The present results align well with those from these widely divergent organisms and strongly suggest common molecular mechanisms involved in unreduced gamete formation.

## Background

Sexual reproduction of eukaryotes requires meiotic cell division which halves the chromosome number in gametes through a single DNA replication followed by two successive chromosome divisions. The first phase, meiosis I, involves homologous chromosome pairing, chiasmata formation and recombination followed by chromosome segregation without sister chromatid disassociation to reduce the number to half (reductional division). The second phase, meiosis II, resembles mitosis and involves segregation of sister chromatids (equational division) and the formation of haploid gametes. This meiotic pathway involves highly conserved and coordinated processes and any deviations from it may lead to gametes with abnormal chromosome constitutions [1–3]. Meiotic restitution is one such deviation in which the germ cell divides only once and nuclear restitution leads to the formation of unreduced (2*n*) gametes [1, 4]. Unreduced gametes occur relatively frequently in interspecific or intergeneric hybrids [5] and less frequently in nonhybrid species [3]. Unreduced gametes are a driving force in the widespread polyploidization and speciation of flowering plants [1, 6].

Two major types of meiotic restitution have been recognised in the formation of unreduced gametes. First division restitution (FDR) results from failure of reductional chromosome segregation during meiosis I followed by normal equational division of sister chromatids during meiosis II. Alternatively, second division restitution (SDR) occurs when a normal reductional division at meiosis I is followed by failure of equational division at meiosis II [1, 4]. In both cases, dyads carrying an unreduced number of chromosomes are the main end products of meiosis.

Unreductional meiosis in plants is often associated with asynapsed univalents at meiosis I. In some polyhaploid wide hybrids in the bread wheat tribe *Triticeae* the univalents often fail to undergo poleward segregation during anaphase I, and the meiocyte enters normal meiosis II, generating unreduced FDR gametes [5, 7]. Asynaptic mutants in *A. thaliana*, rice and maize have shown the same phenomenon [8–10]. In many cases, the univalents undergo precocious separation of sister chromatids (PSSC) resulting in equational division at the first and only meiotic division**.** Cells then exit meiosis and so dyads with unreduced gametes are formed [11–13]. This process has been designated ‘single division meiosis’ (SDM) by Matsuoka and Nasuda [11] or ‘mitotic-like meiosis’ by Zhang and co-workers [13]. SDM has been shown to co-occur with either FDR or SDR in individual *Triticeae* hybrids [13–15]. Meiotic nonreduction through SDM has also been reported in a non-hybrid synaptic mutant of *Paspalum jesuiticum* [16].

Another cytological pathway leading to meiotic restitution was reported by Lim and co-workers [17] in *Lilium* interspecific hybrids with incomplete homology between the parental genomes. Some chromosomes paired to form bivalents that usually segregated reductionally at anaphase I, while unpaired univalents divided equationally. This pathway combined features of both FDR and SDR and was called ‘índeterminate meiotic restitution’ (IMR) [17].

Hybrids with closely related genomes can also produce 2*n* gametes following nearly complete chromosome pairing and reductional segregation of homoeologs during meiosis I, as reported in intra-sectional diploid *Lilium* hybrids [18]. In that case, failure of cytokinesis after anaphase II led to 2*n* gametes by SDR. Failure of equational division of sister chromatids at anaphase II can also generate 2*n* gametes by SDR [4].

The present study explores the mechanisms of meiotic nonreduction leading to functional unreduced gametes in a wide *Trifolium* (clover) hybrid. *Trifolium* L. section *Trifoliastrum* includes tetraploid white clover (*T. repens*, 2*n* = 4*x* = 32) an important forage species adapted to temperate climates world-wide. The section also includes closely related species that together form a diploid-polyploid species complex [19–21]. Two members of this complex, *T. occidentale* (2*x* and synthetic 4*x*) and *T. ambiguum* (Caucasian clover, 2*x*, 4*x*, 6*x*) are more drought tolerant than white clover and carry other useful traits that would improve the environmental range of this key forage species [22, 23]. *T. ambiguum* and *T. occidentale* can be crossed using embryo rescue to form partially fertile hybrids [20, 21, 24]. A 4*x* hybrid (hybrid-33) between 6*x T. ambiguum* and 2*x T. occidentale* was found to be partially fertile through the formation of unreduced gametes [20]. Hybrid-33 was a key breeding parent as it was inter-fertile with both *T. repens* and *T. ambiguum*, generating breeding populations with potential for introgressing drought tolerance and other agronomically useful traits into white clover or, alternatively, from white clover and *T. occidentale* into Caucasian clover.

This study explores microsporogenesis in hybrid-33 and its progeny using 5S and 18S rDNA probes, the latter detecting 18S-26 rDNA in the nucleolus organizer region (NOR), to identify and trace species-specific chromosomes across generations [25]. The results indicated that unreductional meiosis leading to 2*n* gametes occurred when there was a high frequency of asynapsis at meiosis I and that reductional meiosis was restored in progeny where asynapsis frequencies were low. These results are consistent with evidence largely from wheat and its relatives and some insects and are now extended to hybrids in the dicotyledonous plant genus *Trifolium*.

## Results

**Fig. 1:**
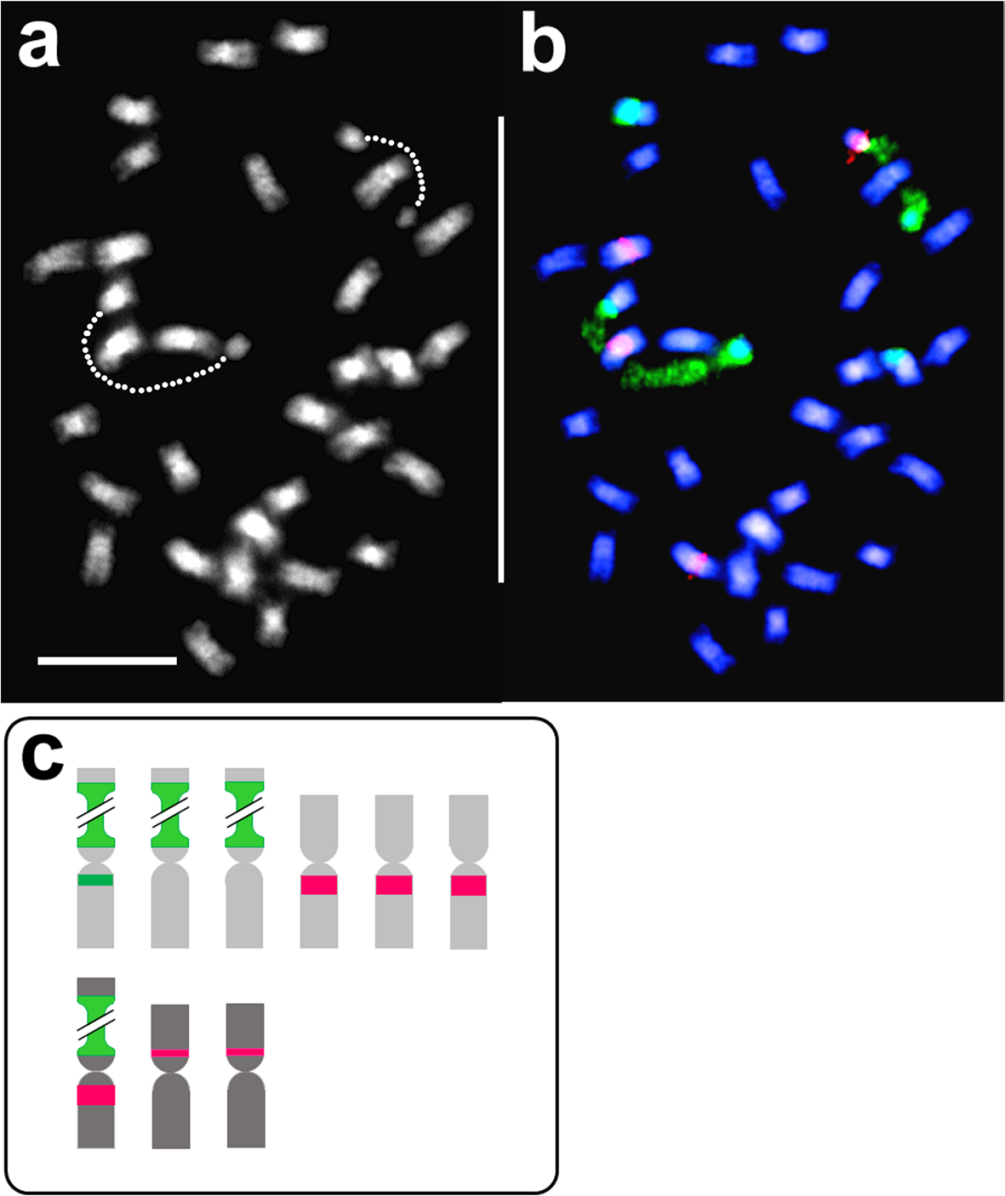
Somatic chromosomes of hybrid-33. **a**. DAPI stained metaphase cell; dotted lines show decondensed NORs. **b**. Same cell as in **a** after FISH with 5S rDNA (red) and 18S–26S(green) rDNA signals. **c**. Diagrammatic representation of 5S (red) and 18S–26S (green) rDNA carrying marker chromosomes from *T. ambiguum* (light grey) and *T. occidentale* (dark grey) genomes

FISH mapping of 5S and 18S–26S rDNA loci [25] of hybrid-33 (2*n* = 4*x* = 32) confirmed the presence of three genomes of *T. ambiguum* and one of *T. occidentale* [20] (Fig. 1). Nine marker chromosomes with rDNA loci in hybrid-33 (Fig. 1b, c) were useful in analysing the genomic constitution of progeny plants. One of the three *T. ambiguum* NOR-chromosomes displayed an additional minor 18S–26S rDNA signal proximally on the long arm (Fig. 1b, c).

### Male meiosis in hybrid-33

**Fig. 2:**
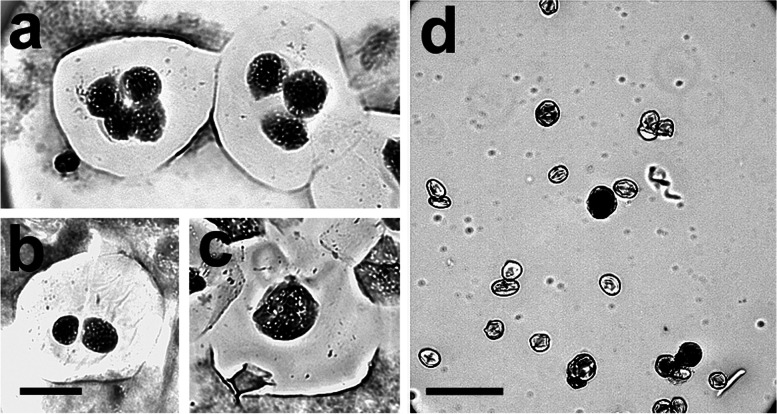
Male meiotic products of hybrid-33. **a**. a tetrad and triad in the same microscope field; **b**. a dyad; **c**. a monad; **d**. aceto-carmine stained pollen grains. Sterile pollen grains are small, wrinkled and unstained. Scale bar in **b** is 10 μm and applies also to **a** and **c**. Scale bar in **d** is 100 μm

Extensive hand pollination of hybrid-33 (with the parent species and white clover) produced no seed, suggesting complete sterility, but open pollination with the same pollen sources produced a few seeds. Because of the very low female fertility, we were able to analyse only male meiosis in pollen mother cells (PMCs). Post-meiotic products of hybrid-33 observed in chromosome preparations included tetrads, triads, dyads and monads (Fig. 2a, b, c). When treated with 1% acetocarmine most pollen grains were unstained and collapsed (sterile), but 2% (16 in a sample of 800), were notably large, smooth and stained, indicating fertility (Fig. 2d).

**Fig. 3:**
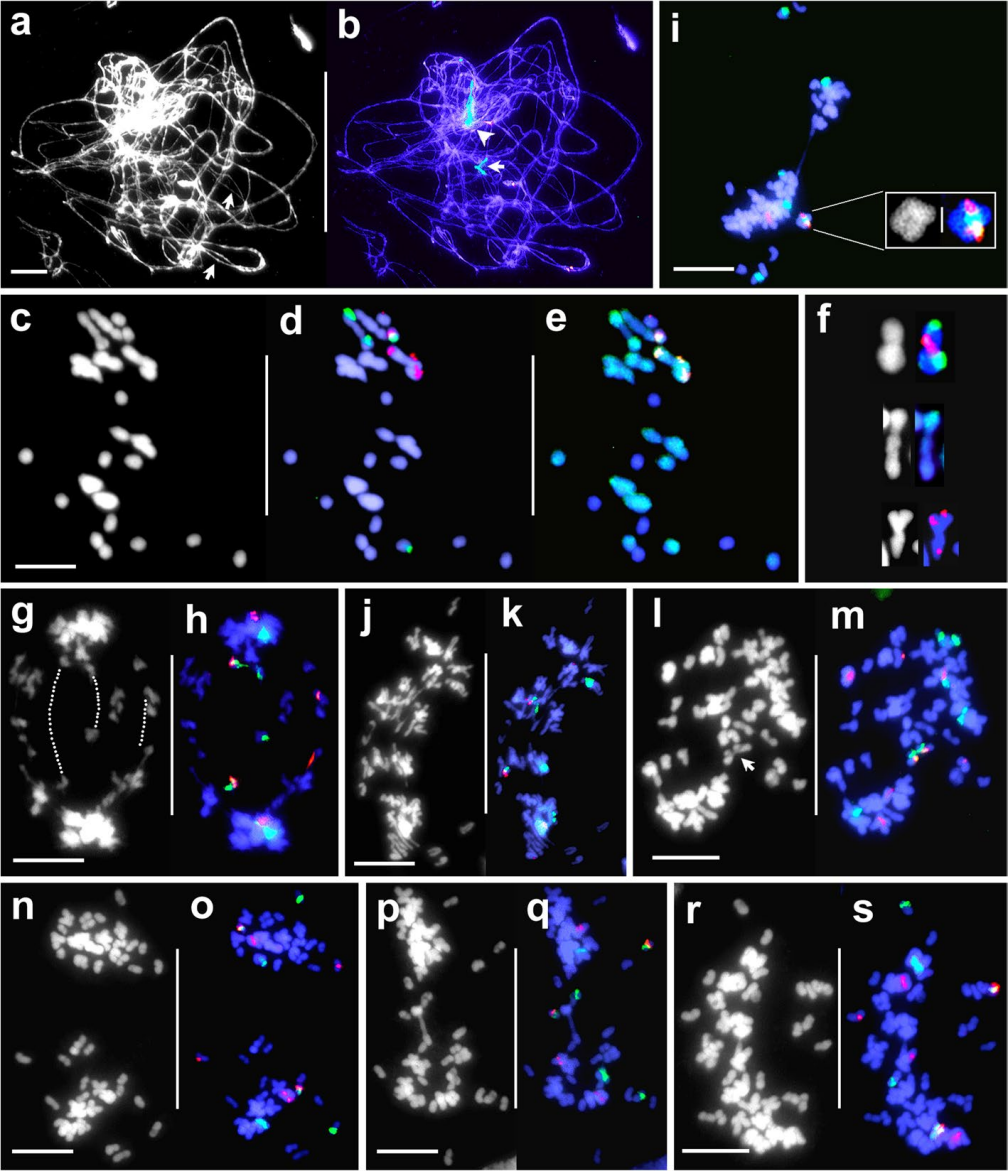
Meiosis in hybrid-33 (pachytene to metaphase II) after FISH mapping with 5S (red) and 18S–26S (green) rDNA sequences and sequential GISH using total genomic *T. ambiguum* DNA (green). Chromosomes are counter stained with DAPI (grey scale or blue). **a-b**. A pachytene cell after DAPI staining (**a**, grey scale) and FISH (**b**). Thickness differences among chromatin threads represent variability in the number of synapsed chromosomes or chromatids. In **a**, *arrows* indicate irregular synapsis; **b**. shows an unpaired *T. occidentale* NOR chromosome (*arrow*) and three synapsed *T. ambiguum* NOR chromosomes (*arrowhead*). **c-e**. A metaphase I cell; **c.** DAPI-stained (grey scale), **d**-**e**. FISH and sequential GISH, respectively. Synaptic anomalies are apparent as multivalents and scattered univalents. **f**. Three examples of anomalous pairing selected from other metaphase I cells. A homoeologous bivalent between *T. occidentale* and *T. ambiguum* NOR chromosomes after DAPI staining and FISH (top), a nonhomologous intergenomic trivalent after GISH (centre), and a homologous trivalent of 5S rDNA carrying *T. ambiguum* chromosomes (bottom). **g-h.** Anaphase I Type 1 cell after DAPI staining (**g** grey scale) and FISH (**h**) showing complete poleward segregation of chromosomes apart from several sister chromatid laggards. Guidelines in **g** denote PSSC in *T. occidentale* and *T. ambiguum* univalents identifiable in **h**. **i**. AI-Type1 cell after FISH showing a *T. occidentale* NOR chromosome with a translocated additional 5S rDNA signal (enlarged in the inset, DAPI stained, left, FISH, right). **j-k**. AI-Type2 cell after DAPI staining (**j**) and FISH (**k**) showing partial poleward chromosome segregation. **l-m**. AI-Type3 cell after DAPI staining (**l**) and FISH (**m**) with intact chromosomes and separated sister chromatids, showing complete failure of anaphase I segregation. The *arrow* in **l** shows sister chromatid separation of a *T. occidentale* derived NOR univalent (identifiable in **m**). **n-o.** MII-Type1 cell after DAPI staining (**n**) and FISH (**o**) showing daughter nuclei with chromosomes surrounded by precociously separated sister chromatids. **p-q.** MII-Type2 cell after DAPI staining (**p**) and FISH (**q**) showing partial anaphase I movement. **r-s.** MII-Type3 cell after DAPI staining (**r**) and FISH (**s**) showing no anaphase I movement. Intact chromosomes and precociously separated sister chromatids are visible. Scale bar: 10 μm

Around 230 PMCs from hybrid-33 at different meiotic stages were analysed. Synaptic irregularities were observed as early as at pachytene (Fig. 3a, b), where synapsis discontinuities and multivalent formations were frequently observed. In 80 Giemsa stained and FISH-GISH treated PMCs analysed at metaphase I, univalents were invariably the major feature, averaging 12.5 per cell (approximately 39% of the genome). There were also mean numbers of 7 bivalents and 1.8 multivalents per cell. FISH and sequential GISH analyses in 36 metaphase I cells showed that only four cells (about 11%) displayed the expected pairing pattern where two genomes of *T. ambiguum* paired with each other to form 8 bivalents while those from the third genome of *T. ambiguum* and the single *T. occidentale* genome were unpaired as 16 univalents. In the remaining 32 cells, univalent numbers ranged from 5 to 17 and were from both parental genomes. Cells in which the univalents represented most of the single *T. occidentale* genome were frequently encountered. Paired chromosome entities in these 32 metaphase I cells were bivalents and multivalents (mostly trivalents) formed through inter- or intra-genomic pairing without any specific patterns (Fig. 3c-f). Typical metaphase I mid-zone congression of chromosomes was rarely encountered. Instead, paired entities were frequently loosely aligned at the equatorial plate while univalents, irrespective of genomic origin, were always randomly scattered throughout the cell (Fig. 3c-e).

Of 70 PMCs analysed at anaphase I, 43 (approximately 62%) showed bivalent halves and partners of multivalents (with clear discrimination of sister chromatids in both arms) segregating reductionally towards the opposite poles as in normal meiosis I. Within the same nucleus, the univalents from both genomes invariably displayed precocious separation of sister chromatids. Their segregation resulted in a mitosis-like equational division, as evidenced by the presence of marker sister chromatids at opposite poles (Fig. 3g-h). In most cases, sister chromatids displayed slower segregational movement than partners of paired entities. This asynchronicity resulted in the formation of chromatid laggards in almost all PMCs (Fig. 3g-h). The mean number of precociously separated chromatids at the end of anaphase I in 30 well spread cells was found to be 26, which is compatible with the mean number of 12.5 univalents per cell observed at metaphase I. Based on variable anaphase chromosome movement, we classified PMCs at anaphase I in three groups. PMCs with chromosomally segregated defined poles, described above, were included in AI-Type1 (62%). The remaining 38% of PMCs (27/70) either displayed partial chromosome movement towards anaphase I poles (AI-Type2, Fig. 3j-k) or displayed no chromosomal movement (AI-Type3, Fig. 3l-m). In AI-Type2 cells, the blurred chromosomal boundary between the two daughter nuclei was occupied by laggards including precociously separated sister chromatids (Fig. 3j-k). Despite the complete lack of anaphase I movement in AI-Type3 cells, paired entities experienced proper disjunction of their halves and sister chromatids disjoined precociously in almost all the univalents (Fig. 3l-m). As in anaphase I, PMCs in metaphase II could be classified in three types, viz., MII-Type1 (Fig. 3n,o), MII-Type2 (Fig. 3p,q) and MII-Type3 (Fig. 3r,s). In all three PMC types, partners of paired entities were generally organised in the equatorial zone while precociously separated sisters displayed a lack of congression (Fig. 3n-s).

**Fig. 4:**
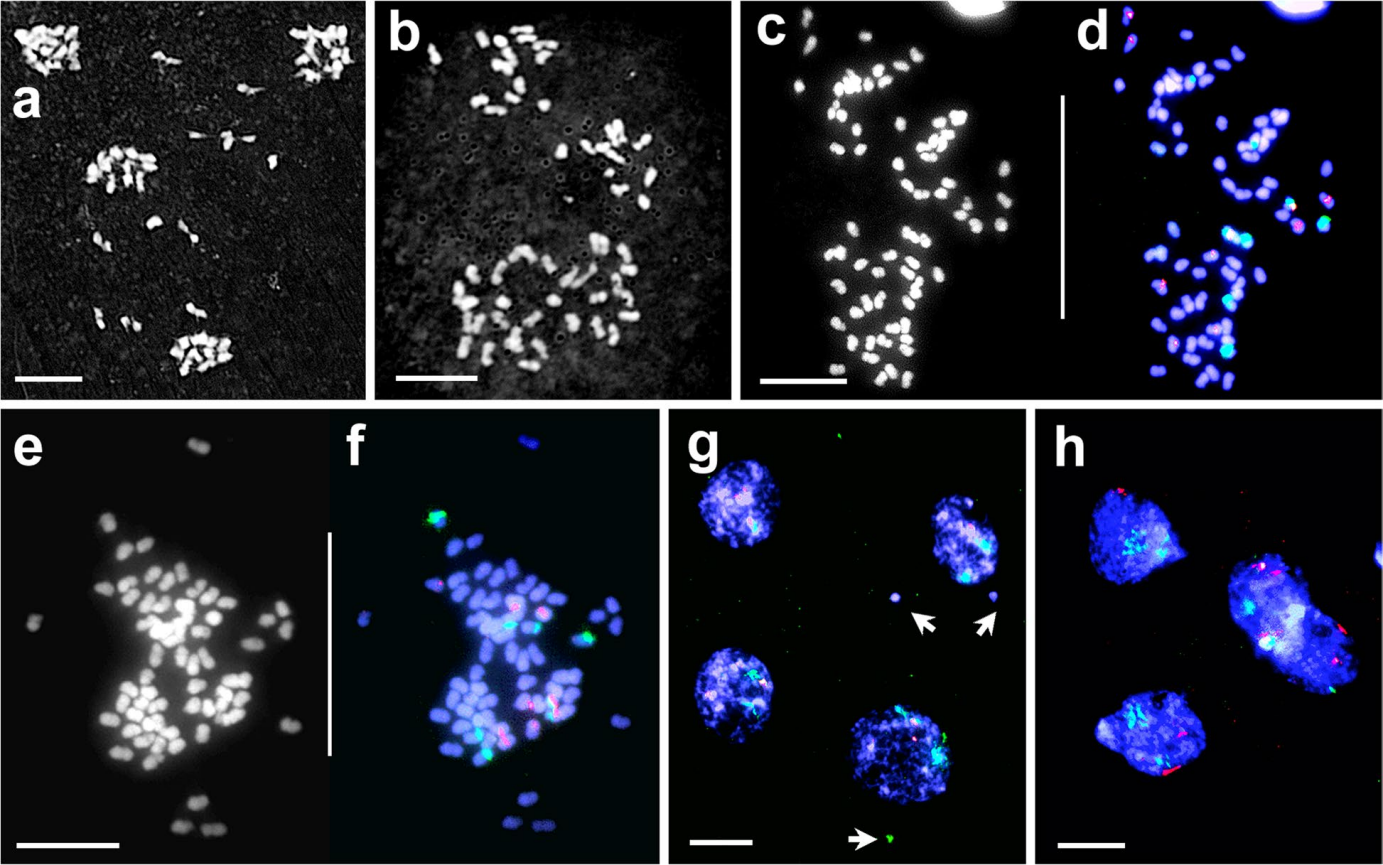
Meiosis in hybrid-33 (anaphase II to telophase II). **a**. Inverted Giemsa anaphase II cell with four daughter nuclei and several laggard chromatids. **b**. Anaphase II cell with three daughter nuclei (one unreduced). **c-d**. Anaphase II cell after DAPI staining (**c**) and FISH (**d**) with two unreduced daughter nuclei due to failed anaphase II segregation. **e-f**. Anaphase II cell after DAPI staining (**e**) and FISH (**f**) with 8*x* = 64 chromosomes after two successive anaphase segregation failures. **g**. Telophase II cell after FISH, with four daughter nuclei and several laggard chromatids (*arrows*). **h**. Telophase II cell after FISH, with three daughter nuclei. Scale bar: 10 μm

At anaphase II, normal disjunction of sister chromatids by separation of sister kinetochores in half bivalents and partners of multivalents was consistently observed in all the analysed PMCs. In most PMCs, newly separated sister chromatids moved equationally towards opposite poles while precociously separated chromatids migrated randomly. Four daughter nuclei with reduced chromosome numbers were formed but the asynchronous movements of chromatid laggards led to unbalanced chromosome contents at telophase II (Fig. 4a, g). In cases where anaphase II segregational movement failed in one of the two nuclei derived from AI-Type1 PMCs three daughter nuclei were formed, two near haploid (2*x*; reduced) and one near diploid restituted nucleus (4*x*; unreduced) (Fig. 4b, h). Segregational failure in both nuclei was observed to produce two unreduced nuclei (Fig. 4c-d*.*). Two unreduced daughter nuclei at the end of anaphase II could also have formed through normal segregation from restituted AI-Type3 PMCs (Fig. 4c-d). A second failure of sister chromatid segregation at anaphase II, involving AI-Type3 PMCs, produced rarely observed 8*x* gametes (chromosome count 64) (Fig. 4e-f).

### Cytogenetics of first-generation progeny plants (33OP-1 and 33OP-14)

**Fig. 5:**
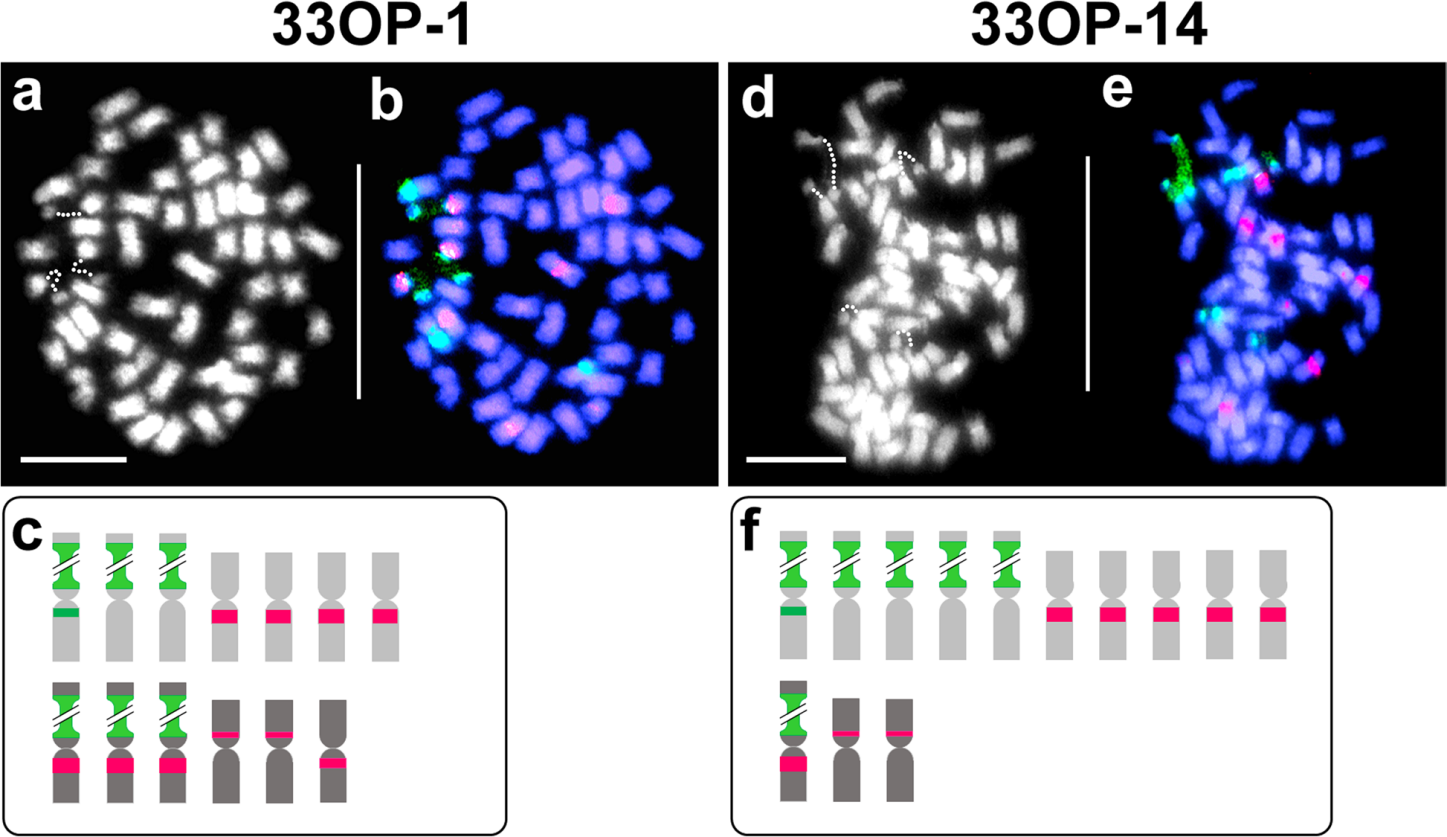
Somatic chromosomes of hybrids 33OP-1 (left column) and 33OP-14 (right column) after DAPI staining (**a**, **d**) and FISH (**b**, **e**). Diagrammatic representation of rDNA (red 5S; green 18S–26S) marker chromosomes derived from *T. ambiguum* (light grey) and *T. occidentale* (dark grey) are shown in **c** and **f**. Decondensed NORs, visible after FISH in **b** and **e**, are depicted by dotted lines in **a** and **d**. Scale bar: 10 μm

Chromosome counts for two open-pollinated (OP) progeny plants were confirmed as 51 for 33OP-1 (Fig. 5a-c) and 56 for 33OP-14 (Fig. 5d-f) as reported earlier [20]. In 33OP-1, thirteen 5S and 18S–26S rDNA marker chromosomes (Fig. 5c) revealed that an aneuploid (2*n* = 35) unreduced female gamete from hybrid-33 had been fertilized by a normal (*n* = 16) male gamete from white clover (Fig. 5c). In addition to the expected unreduced chromosomal constitution, the female gamete had three extra marker chromosomes- one with 5S rDNA from *T. ambiguum*, one with colocalised 5S and 18S–26S rDNA loci and one with a minor 5S rDNA locus, the last two from *T. occidentale* (Fig. 5c). Hybrid-33OP-14 also displayed 13 marker chromosomes (Fig. 5d-f). Analysis indicated that the female gamete from hybrid-33 was again unreduced and aneuploid. The pollinator was either 4*x* or 6*x T. ambiguum*. There were two *T. occidentale* descended chromosome with minor 5S FISH signals instead of one expected via the female gamete (Fig. 5f).

### Restoration of reductional meiotic division in 33OP-1 and 33OP-14

**Fig. 6:**
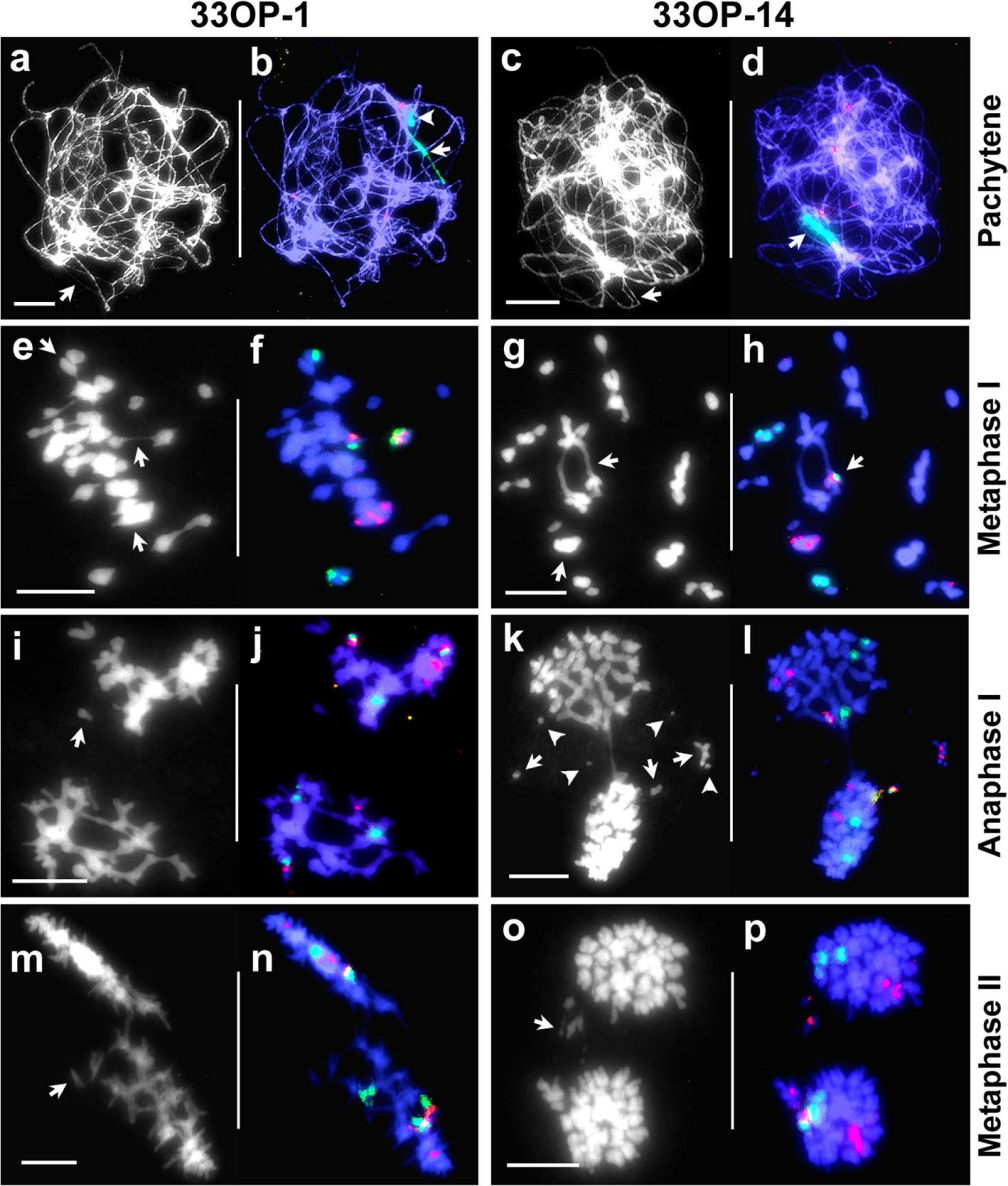
Meiosis (pachytene to metaphase II) in hybrids 33OP-1 (left column) and 33OP-14 (right column). **a-d.** Pachytene cells after DAPI staining (**a**, **c**) and the same cells after FISH (**b**, **d**). In **a** and **c** the arrows show irregular synapsis. In **b**, NOR-carrying chromosomes from *T. ambiguum* (*arrow*) and from *T. occidentale* and *T. repens* (*arrowhead*) form separate trivalents. In **d** the arrow indicates multivalent formation involving NOR chromosomes from both parental genomes. **e-h**. Metaphase I cells after DAPI staining (**e**, **g**) and the same cells after FISH (**f**, **h**). *Arrows* in **e** indicate abnormal pairing of marker chromosomes visible in **f**. The *arrow* in **g** indicates a nonhomologous multivalent involving the *T. occidentale* NOR chromosome (*arrow* in **h**). **i-l.** Anaphase I cells after DAPI staining (**i**, **k**) and the same cells after FISH (**j**, **l**) showing several laggards, including precociously separated sister chromatids (*arrows*) and chromosomal fragments (*arrowheads*). **m-p.** Metaphase II cells after DAPI staining (**m**, **o**) and the same cells after FISH (**n**, **p**) with sister chromatid laggards (*arrows* in **m**, **o**). Scale bar: 10 μm

In both plants, synaptic irregularities, including abnormal marker chromosome associations and weak or incompletely synapsed axes appeared as early as pachytene (Fig. 6a-d). Univalents were a consistent feature at metaphase I in both plants, but in lower numbers than hybrid-33. An average of 3.9 univalents per PMC (38 cells; 7.6% of the genome) were encountered in 33OP-1. The remaining 47.1 chromosomes (92.4%) were involved in synapsis, forming bivalents to multivalents (Fig. 6e-f). In 33OP-14 there was an average of 2.6 univalents (22 cells; 4.7% of the genome) (Fig. 6g-h), while 53.4 chromosomes (95.3%) formed bivalents and multivalents. Analysis of FISH markers revealed homologous, homoeologous and non-homologous pairing in both hybrids.

**Fig. 7:**
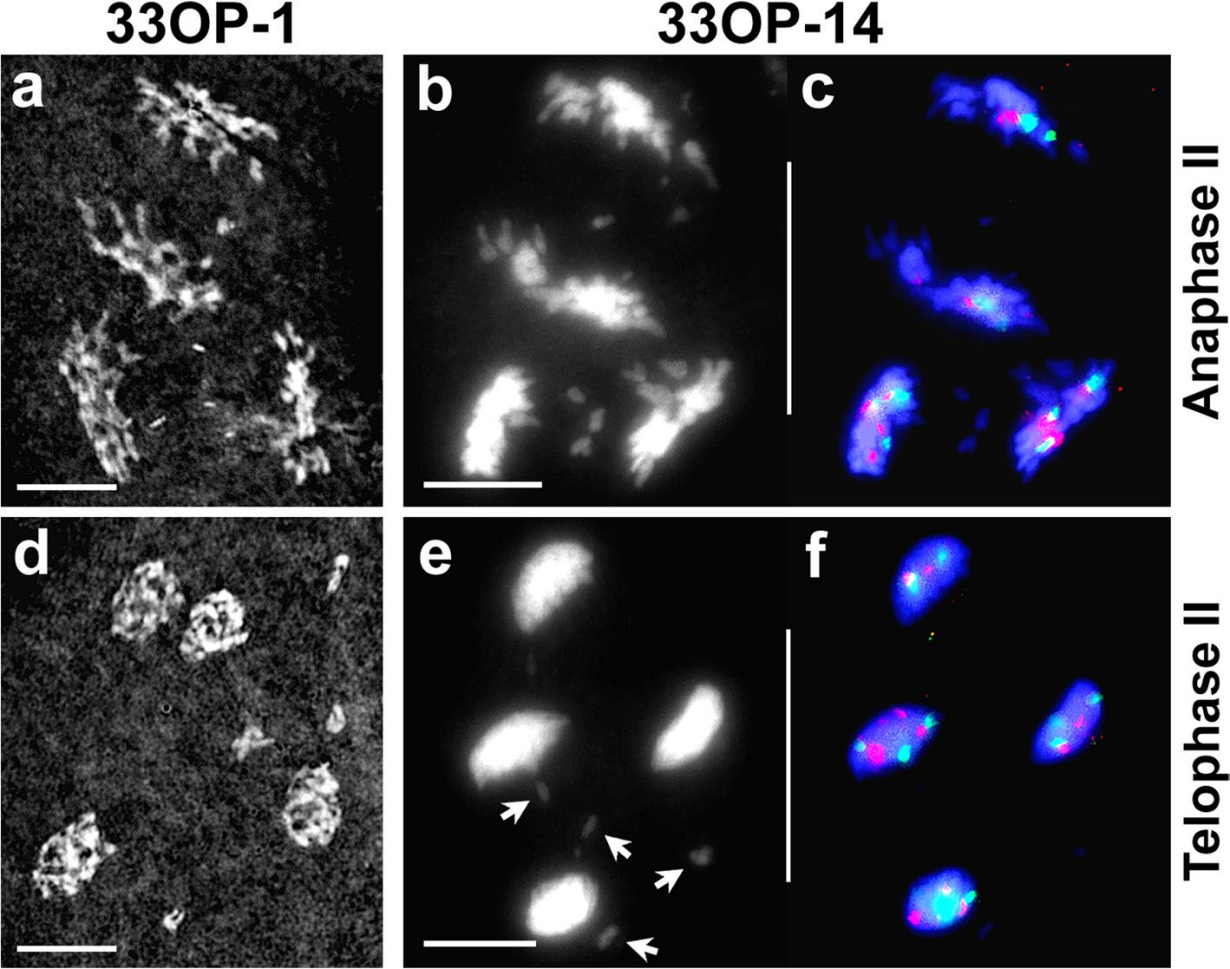
Meiosis (anaphase II to telophase II) in hybrids 33OP-1 (left column) and 33OP-14 (right column). The presence of four daughter nuclei in all the cells indicates reductional division. **a**. Giemsa stained (inverted) anaphase II cell showing four daughter nuclei with several laggards. **b-c**. Anaphase II cell after DAPI staining (**b**) and the same cell after FISH (**c**) showing four daughter nuclei with laggard chromatids. **d**. Giemsa stained (inverted) telophase II cell with a few laggards. **e-f**. Telophase II cell after DAPI staining (**e**) and FISH (**f**) with several laggards (*arrows* in **e**). Scale bar: 10 μm

At anaphase I in both plants, reductional division occurred with complete disjunction of paired entities and defined poleward segregation (Fig. 6i-l). However, all the univalents underwent precocious separation of sister chromatids, displaying asynchronicity in segregation and occasionally forming laggards (Fig. 6i-l). Unequal segregation of FISH signals was observed in most of the anaphase I and metaphase II meiocytes, reflecting synaptic and segregational irregularities **(**Fig. 6j, l, n, p**).** At anaphase II, equational disjunction and segregation of sister chromatids was routinely observed (Fig. 7a-c), giving rise to four daughter nuclei with reduced chromosome numbers. Neither plant showed any PMCs with restitution nuclei. However, chromatid laggards were always present, presumably from the precociously separated chromatids, and were likely to have been eliminated in telophase II (Fig. 7d-f**)**.

**Fig. 8:**
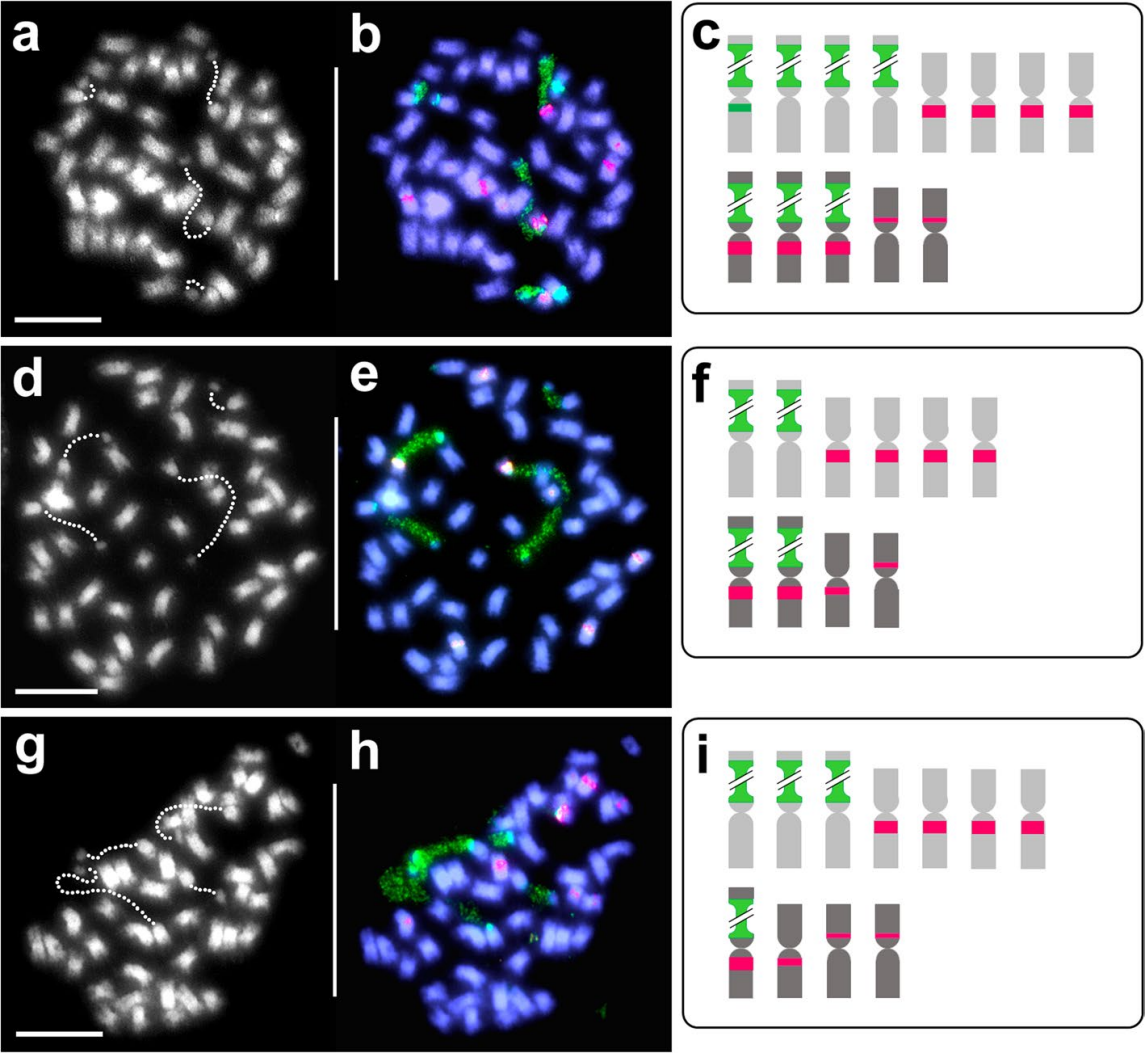
Somatic chromosomes of three self-progeny plants of hybrid-33OP-1 after FISH and with diagrammatic representation of the marker chromosomes: 33OP-1-self-3 (**a**-**c**); 33OP-1-self-6 (**d**-**f**) and 33OP-1-self-13 (**g**-**i**). For other details, refer Figs. 2 and 5. Scale bar: 10 μm

As reported earlier [20], 33OP-1 produced selfed seeds. Three partially fertile selfed progeny plants from 33OP-1, viz., 33OP-1-self-3, − 6 and − 13 were cytologically investigated. The chromosome counts of 47 for 33OP-1-self-3 and -6, and 45 for 33OP-1-self-13 confirmed the restoration of reductional meiosis and the formation of functional gametes of both sexes. The inheritance of different numbers of marker chromosomes among the three selfed progeny plants (Fig. 8) revealed that significant meiotic irregularities nevertheless persisted in hybrid-33OP-1.

## Discussion

The partial fertility of hybrid-33 via unreduced (and aneuploid) female gametes was discovered after open pollination [20]. We could not analyze female gametogenesis in this hybrid, but we did a thorough analysis of male gametogenesis. This confirmed that nonreductional meiosis led to the formation of unreduced male gametes, and that gametogenesis in both sexes of hybrid-33 probably underwent similar meiotic pathway deviations leading to nonreduction.

A lack of homology between the parental genomes in interspecific F_1_ plant hybrids induces meiotic restitution leading to unreduced gamete formation [26, 27]. Amphihaploid and polyhaploid wide hybrids in the *Triticeae* showed almost complete lack of meiotic pairing [28–31]. Such hybrids produced predominantly unreduced gametes through FDR or single division meiosis, and displayed complete restitution, often referred to as FDR-type sensu *strictu* [26]. Cai and co-workers [28] referred to such meiotic restitution as ‘haploidy-dependent’ unreductional meiotic cell division. In contrast, hybrids with partial homology among parental genomes displayed meiotic synapsis and formed bivalents and univalents during meiosis I [32, 33]. In such cases, the meiotic pathway favored reductional division and tetrad formation and almost eliminated unreduced gamete formation [29, 34]. A study of a hexaploid wheat line with temperature-dependent asynapsis showed that meiotic non-reduction would be better described as ‘asynapsis-dependent’ rather than ‘haploid-dependent’ [35]. Other workers have preferred ‘univalent-dependent’ [12, 26].

**Fig. 9:**
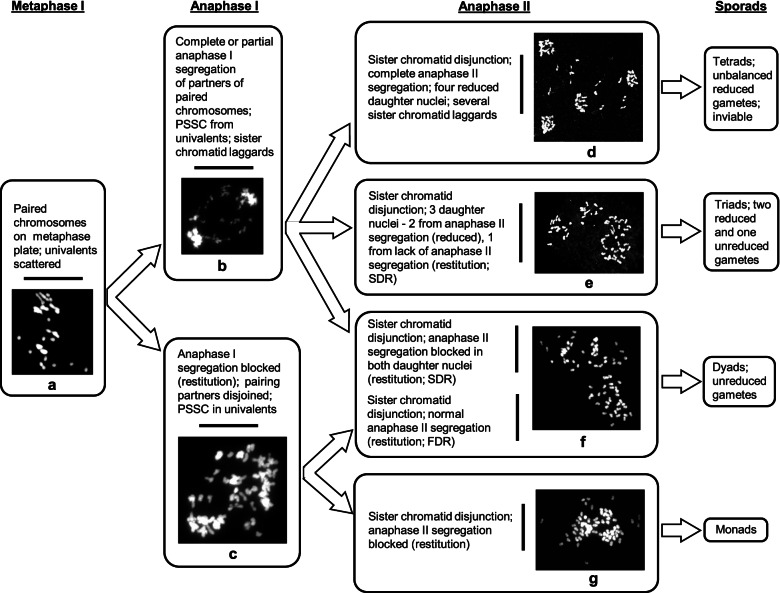
Flow diagram showing the meiotic pathways leading to unreduced gamete formation in hybrid-33

In hybrid-33, meiocytes displayed an average of 12.5 univalents and several paired entities in meiosis I (Figs. 3 c-f, 9a). At meiosis I, univalents displayed equational separation of sister chromatids while paired entities showed reductional division. (Figs. 3g-h, 9b**)**. This resembled IMR as reported in Asiatic lily hybrids [17] except that, in hybrid-33, all the meiocytes entered both meiotic phases. The majority of meiocytes underwent a normal second meiotic phase and produced four reduced daughter nuclei (Figs. 4a, 9d) leading to tetrad formation. Pollen grains developed from such tetrads were sterile (Fig. 2d) due to genomic imbalances resulting from segregational irregularities [29, 30].

Restitution in hybrid-33 occurred in either or both of the two meiotic phases. In the majority of meiocytes, two daughter nuclei were formed in anaphase I as described above (Fig. 9b). Subsequently, while one daughter nucleus went through normal meiosis II, the other one underwent restitution so that a triad was formed with one unreduced and two reduced gametes (Fig. 9e). If such restitution occurred in both the daughter nuclei then both would have been unreduced (Fig. 9f) leading to dyad formation (i.e., effectively SDR within the parameters of IMR). Alternatively, a smaller frequency of meiocytes attained restitution during meiosis I (Fig. 9c). Here, univalents went through disjunction of their sisters while partners of paired entities disjoined. However, chromosomal segregation failed during anaphase I, and thus restitution occurred in the first meiotic phase. As these meiocytes entered meiosis II, sisters from intact chromosomes segregated normally while precociously separated sisters segregated randomly. The unreduced dyads thus formed (Fig. 9f) were attributable to FDR (within the parameters of IMR). Dyads formed in hybrid-33 could therefore be achieved through pathways both SDR and FDR. A very small frequency of meiocytes of hybrid-33 which were restituted during meiosis I, also failed anaphase II segregation (Fig. 9g) and developed into monads (Fig. 2c) with a chromosomal constitution of 4*n*. Similar results were reported in a polyhaploid from triticale [30] and in *Hierochloe odorata* (holy grass) [36].

Faithful segregation of meiotic chromosomes, is normally ensured through processes involving cohesion of sister chromatids, mediated by a cohesion complex involving meiosis-specific cohesin proteins, including Rec8, and its stepwise removal by a protease enzyme, separase [37]. At meiosis I, Rec8 cohesion holds together sister chromatids during bivalent formation, promoting monopolar orientation of sister kinetochores and their attachment to spindle fibres emanating from the same pole [8, 10, 38]. At anaphase I, cleavage of Rec8 occurs in the chromosome arms but cohesion is retained at the centromere allowing reductional segregation of the homologous partners to opposite poles without sister chromatid dissociation [39]. At anaphase II, the second step of cohesion removal releases kinetochores, promoting bipolar orientation and attachment to spindle fibres emanating from opposite poles and leading to equational separation of sister chromatids. Recent studies have shown, however, that asynapsed univalents at meiosis I often show weakened centromeric Rec8 cohesion [8, 10, 40], leading to bipolar rather than monopolar orientation of sister kinetochores [41]. Consequently, sister kinetochores attach to opposite poles [8, 10, 28, 41–43]. If the cohesion is removed early, at the transition of metaphase I to anaphase I, univalents display premature separation of sister chromatids and undergo equational division [28, 33, 44]. Alternatively, if the removal of cohesion is incomplete or the removal continues during anaphase I, the spindle collapses due to opposing forces between the spindle microtubules and the remnant kinetochore cohesion, laggards occur, and an unreduced restitution nucleus is generated [28, 29, 44, 45].

In hybrid-33, 62% of anaphase I cells displayed complete segregation, with polar clusters of disjoined bivalents and multivalents with intervening laggards from precociously but asynchronously segregating univalent sister chromatids (AI Type 1, Figs. 3g-h, 9b). This was consistent with the first alternative above, i.e., aberrant bipolar orientation of sister kinetochores in univalents coupled with loss of centromeric cohesion in early anaphase I [28, 44]. A smaller frequency of hybrid-33 PMCs showed a complete failure of anaphase I segregation, resulting in a completely restituted nucleus (AI-Type3, Fig. 3 l-m). This was consistent with the alternative model where the removal of centromeric cohesion in univalents was delayed until late anaphase I, provoking spindle collapse and a restituted nucleus [28, 33, 44–46]. The intermediate AI- and MII-Type 2 phenotypes (Figs. 3 j-k, p-q) observed in a few PMCs are difficult to classify as they may have several possible causes, including incomplete spindle collapse [44].

Paliulis and Nicklas [47] demonstrated that grasshopper chromosomes manually transferred between meiosis I and meiosis II spindles segregated as though they were on the original spindle. Therefore, normal segregational behaviour of meiotic chromosomes was an intrinsic property of the chromosomes themselves and not a function of the spindle or cytoplasm. The simultaneous equational division of univalents and reductional division of bivalents and multivalents on the same spindle, as observed in this and other studies, can thus be understood. As noted earlier, variability in the dissolution of centromeric cohesion in univalents during meiosis I may lead to segregational failure [44, 45]. Several studies in the *Triticeae* and in grasshoppers have shown that the greater the number of univalents, the greater the chance of spindle collapse leading to meiotic restitution and unreduced gametes [29, 34, 44]. Hybrid-33 had a high number of univalents at metaphase I (an average of 12.5 among 32 chromosomes) and so the unreduced gamete production was consistent with these observations. Notable also was the restoration of reductional division in the two progeny plants of hybrid-33 which had low numbers of univalents. Hybrid-33OP-1 incorporated a new *T. occidentale* subgenome from *T. repens* [20] while 33OP-14 incorporated new *T. ambiguum* sub-genomes from the male parent, adding to the potential synapsis with *T. occidentale* and *T. ambiguum* sub-genomes already present in 2*n* gametes from hybrid-33. The cumulative effect of these enhanced synaptic potentials was low univalent numbers at metaphase I. 33OP-1 had an average of 3.9 univalents (chromosome count 51) and 33OP-14 had 2.6 univalents (chromosome count 56). Additionally, despite premature separation of sister chromatids in univalents and some laggard formation at anaphase I, neither plant showed any PMCs with restitution nuclei. These results further support the observation that higher numbers of univalents at metaphase I increase the likelihood of unreductional meiotic cell division and 2*n* gamete production, and further justify the terms ‘asynapsis-dependent’ or ‘univalent-dependent’ unreductional meiotic cell division [27, 29, 34, 44].

Karyological changes are frequently observed in progenies of hybrid plants as the results of meiotic irregularities. These changes include polyploidy, aneuploidy and chromosomal structural changes [27, 48]. The FISH marker chromosome analyses of 33OP-1 and 33OP-14 revealed that the 2*n* female gametes from hybrid-33, were not only unreduced but also had markedly different chromosome constitutions (Figs. 5 c,f). Furthermore, the three selfed progeny plants of 33OP-1 (33OP-1-self-3, − 6 and − 13), despite derivation from reductional meiosis, have shown varying chromosome numbers and constitutions (Fig. 8), indicating meiotic irregularities. Such irregularities are attributable to varying numbers of univalents and multivalents, homologous, homoeologous, non-homologous pairing at metaphase I, unequal segregation of multivalents at anaphase I, and chromosome loss through laggards and formation of micronuclei [27, 48]. It was evident that even after reductional division was restored, the progeny plants of hybrid-33 continued to produce karyological variants, many of which were partially fertile [20]. Chromosomal structural rearrangements (translocations, inversions etc) also occur in hybrid progenies [27]. In hybrid-33 and its OP progeny, homoeologous and non-homologous pairing were frequently observed during pachytene and at metaphase I (Figs. 3 a-b, f; 6 a-d). and structurally rearranged chromosomes were observed in anaphase I preparations of hybrid-33 (Fig. 3 i). The positive implications for clover breeding of obtaining fertile progeny from hybrid-33 following interspecific hybridisation and 2*n* gamete formation were discussed by Williams and co-workers [20].

## Conclusions

Meiotic restitution leading to polyploidization through 2*n* gametes is a powerful agent in plant species evolution and crop breeding [48]. The present study has revealed that formation of 2*n* gametes is asynapsis-dependent or univalent-dependent and has thrown further light on the basic mechanisms of unreduced gamete formation in hybrids. This result, previously supported by evidence largely from wheat and its relatives and grasshopper, is also applicable to hybrids from the dicotyledonous plant genus *Trifolium*. The present results align well with those from these widely divergent organisms and strongly suggest common molecular mechanisms involved in unreduced gamete formation.

## Methods

The tetraploid hybrid-33 was a plant produced by hand pollination and embryo rescue by Williams et al. [20]. The female parent (Endura self-12) was derived by self-pollination from a commercially available 6*x T. ambiguum* cultivar (cv. Endura; PGG-Wrightson Seeds, Christchurch, New Zealand). The male parent was a 2*x T. occidentale* plant OCD 44–16 that was generated from seeds of a population from N Spain held by the Margot Ford Germplasm Centre, AgResearch Grasslands, New Zealand (accession OCD 1157 Praia de Lorenzo). This was made available for research purposes only and was originally from a collection made at sea level sites in N Spain under an approved Agreement for the Acquisition of Material for Plant Genetic Resources. Verifications of species identifications were made by WMW. Hybrid-33 was almost completely sterile but did set a few seeds through open pollination (OP) [20]. Two OP progeny plants, hybrids 33OP-1 and 33OP-14, were grown in pots in an insect-free greenhouse and self-pollinated. Three self-progeny plants, 33OP-1-self-3, − 6 and − 13 were studied further.

Somatic chromosome preparations were obtained from actively growing root tips using a flame-drying technique after enzyme maceration as described earlier [25] with minor modifications [49]. Meiotic chromosome preparations were obtained by squashing PMCs from young floral buds after enzymatic maceration. Some slides from each plant were stained with Giemsa solution diluted with Sorensen’s buffer. The DNA probes for FISH experiments, pTr5S (GenBank accession number AF072692), a 596 bp fragment representing a part of 5S rDNA gene family and pTr18S (GenBank accession number AF071069), a 1.8 kb fragment containing almost an entire 18S rDNA sequence from *T. repens*, were labelled with Cy3-dCTP and Fluor-X-dCTP (GE Healthcare, NZ), respectively by nick translation according to the manufacturer’s specifications. Total genomic DNA from *T. ambiguum*, used in GISH experiment on hybrid-33, was labelled with Fluor-X-dCTP.

Double target FISH using the two rDNA probes, post-hybridization washing and counter staining with DAPI on somatic as well as meiotic chromosomes were carried out as described earlier [25]. After recording the images, some of the hybrid-33 meiotic preparations were re-probed for sequential FISH-GISH using labelled total genomic DNA of *T. ambiguum* and 5S rDNA sequences and unlabelled blocking DNA of *T. occidentale* as described by Ansari and co-workers [50]. The slides were screened under a Nikon Microphot-SA epiflurescence microscope. The images were captured through an AxioCam MRm CCD camera (CarlZeiss GmbH, Germany) attached to the microscope and processed with ISIS imaging software (MetaSystems GmbH, Germany). Individual images were composed using Adobe Photoshop software.

## Data Availability

The data sets from this study are available from the corresponding author.

## Abbreviations

2*n* gamete: Unreduced gamete
2*x*: Diploid
4*x*: Tetraploid
6*x*: Hexaploid
DAPI: 4′,6-diamidino-2-phenylindole
F_1_: First hybrid generation
FDR: First division restitution
FISH: Fluorescence in situ hybridization
GISH: Genomic in situ hybridization
IMR: Indeterminate meiotic restitution
ITS: Internal transcribed spacer
NOR: Nucleolus organizer region
OP: Open pollinated
PMC: Pollen mother cell
rDNA: Ribosomal DNA
SDR: Second division restitution
